# Effects of physical activity and sedentary behaviors on cardiovascular disease and the risk of all-cause mortality in overweight or obese middle-aged and older adults

**DOI:** 10.3389/fpubh.2024.1302783

**Published:** 2024-02-12

**Authors:** Yongqiang Zhang, Xia Liu

**Affiliations:** ^1^Department of Cardiovascular Medicine, Hejiang People's Hospital, Luzhou, Sichuan, China; ^2^Department of Clinical Pharmacy, The People's Hospital of Lincang, Lincang, Yunnan, China

**Keywords:** age, BMI, lifestyle, mortality, cardiovascular disease

## Abstract

**Aim:**

The aim of this study was to respectively explore the relationships between physical activity and sedentary behaviors and cardiovascular disease (CVD) and all-cause mortality risk in overweight/obese middle-aged and older patients, and also assess the interaction between physical activity and sedentary behaviors.

**Methods:**

Data of middle-aged and older adults with body mass index (BMI) ≥25 kg/m^2^ were extracted from the National Health and Nutrition Examination Surveys (NHANES) database in 2007–2018 in this retrospective cohort study. Weighted univariate and multivariate logistic regression analyses were used to explore the associations between physical activity and sedentary behaviors and CVDs; weighted univariate and multivariate Cox regression analyses were used to explore the relationships between physical activity and sedentary behaviors with the risk of all-cause mortality. The interaction effect between physical activity and sedentary behaviors on CVD and all-cause mortality was also assessed. We further explored this interaction effect in subgroups of age and BMI. The evaluation indexes were odds ratios (ORs), hazard ratios (HRs), and 95% confidence intervals (CIs).

**Results:**

Among 13,699 eligible patients, 1,947 had CVD, and 1,560 died from all-cause mortality. After adjusting for covariates, patients who had high sedentary time seemed to have both high odds of CVD [OR = 1.24, 95% CI: (1.06–1.44)] and a high risk of all-cause mortality [HR = 1.20, 95% CI: (1.06–1.37)]. Furthermore, being insufficiently active was linked to high odds of CVD [OR = 1.24, 95% CI: (1.05–1.46)] as well as a high risk of all-cause mortality [HR = 1.32, 95% CI: (1.15–1.51)]. High sedentary time and being insufficiently active had an interaction effect on both high odds of CVD [OR = 1.44, 95% CI: (1.20–1.73)] and high risk of all-cause mortality [HR = 1.48, 95% CI: (1.24–1.76)]. Individuals of different ages with/without obesity need to focus on the potential CVD/mortality risk of high sedentary time and low physical activity (all *P* < 0.05).

**Conclusion:**

Reducing sedentary time combined with increasing physical activity may benefit health by reducing both the risk of CVD and all-cause mortality in overweight or obese middle-aged and older adults.

## Introduction

Obesity and being overweight are major public health problems currently affecting the global population. Since 1980, the prevalence of obesity has increased twofold in more than 70 countries and continues to rise in most of them (1). In the United States, the prevalence of adult obesity was as high as 42.4% in 2017–2018 and shows a higher prevalence in the middle-aged and older populations aged 40 years and older (2). Obesity contributes to an estimated 4 million deaths globally, more than two-thirds of which are caused by cardiovascular diseases (CVDs) (1). Studies have indicated that obesity or being overweight increases the risk of CVDs in middle-aged and older adults (3, 4). Therefore, ameliorating the pathophysiologic effects of obesity and being overweight and reducing the associated risk of CVDs and mortality are important propositions that need to be addressed.

Physical activity level is an important modifiable factor affecting human health (5). It has been reported that physical activity is negatively associated with the risk of obesity, CVDs, and mortality (6–8). Both the World Health Organization (WHO) and the American College of Cardiology (ACC)/American Heart Association (AHA) have recommended appropriate physical activity for health promotion (9, 10). In 2020, the WHO updated and provided new recommendations for reducing sedentary behaviors in addition to increasing physical activity, although the current evidence is insufficient to quantify the threshold of sedentary behaviors (10). Sedentary behavior represents the lowest level of the physical activity spectrum and is generally defined as an energy expenditure of <1.5 metabolic equivalents (METs) when sitting or lying down during waking hours. The associations between sedentary behaviors and CVDs and the risk of mortality are influenced by the level of physical activity, and as physical activity increases, the risk of mortality from sedentariness diminishes or even cancels out (11–13). A longitudinal cohort study in Japan showed an interaction between physical activity levels and sedentary behaviors on the risk of all-cause mortality in older adults (14). Nevertheless, in middle-aged and older adults who are overweight or obese, who are at high risk for CVDs, the combined effects of physical activity and sedentary behaviors on CVD and mortality are still unclear.

Herein, we speculated that physical activity and sedentary behaviors have a combined effect on CVD as well as the all-cause mortality risk in middle-aged and older patients who are overweight/obese. We hope the research findings could provide some references for lifestyle intervention and management among those at risk of CVD or mortality.

## Methods

### Study design and participants

In this retrospective cohort study, data on middle-aged and older persons were extracted from the National Health and Nutrition Examination Surveys (NHANES) database between 2007 and 2018. The NHANES surveys are conducted by the National Center for Health Statistics (NCHS) of the Centers for Disease Control and Prevention (CDC) to assess the nutritional and health status of the civilian non-institutionalized population in the United States. It includes a complex, multistage stratified probability sample based on selected counties, blocks, households, and persons within households. The well-trained NCHS professionals conduct interviews in participants' homes, and extensive physical examinations are conducted at mobile exam centers (MECs). More details are available on the NAHENS webpage: https://www.cdc.gov/nchs/nhanes/index.htm.

Initially, 15,076 adults aged 40–79 years with a body mass index (BMI) of ≥25 kg/m^2^ were included. The exclusion criteria were (1) missing information on CVD, sedentary behaviors, or all-cause mortality (*n* = 225) and (2) missing information on the potential covariates, including marital status, education level, smoking, healthy eating index 2015 (HEI-2015) (15), and chronic kidney disease (CKD) (*n* = 1,152). Finally, 13,699 individuals were deemed eligible. The NHANES was approved by the Institutional Review Board (IRB) of the NCHS. The data were anonymized, and all the participants provided informed consent. No ethical approval of our agency's IRB was required since this database was publicly available.

### Assessments of physical activity and sedentary behaviors

Physical activity was converted into MET, which was calculated based on the information collected via the NHANES physical activity questionnaire (PAQ). The corresponding questions were as follows: “In a typical week, do you do any vigorous-intensity sports, fitness, or recreational activities that cause large increases in breathing or heart rate like running or basketball for at least 10 min continuously?” and “In a typical week, do you do any moderate-intensity sports, fitness, or recreational activities that cause a small increase in breathing or heart rate such as brisk walking, bicycling, swimming, or volleyball for at least 10 min continuously?” Energy expenditure (MET·min) = recommended MET × exercise time of corresponding activity (min). According to a previous study, we classified physical activity into two levels: energy expenditure <450 MET·min/week was defined as insufficiently active; otherwise, it was defined as sufficiently active (9).

The information on sedentary behavior was also self-reported through the PAQs, with the question “How much time do you usually spend sitting on a typical day?” In specific, sedentary behavior was defined as the time spent sitting at school or home and getting to and from places, including sitting at a desk, traveling in a car or bus, reading, playing cards, watching television, or using a computer, which does not include time spent sleeping. The cutoff value for sedentary behaviors was 6 h/day, according to a previous study (16).

### Outcomes and follow-up

The study outcomes were CVD and all-cause mortality. CVD was self-reported based on the NHANES multiple-choice question (MCQ) with the question: “Have you ever been told you had (congestive) heart failure, coronary heart disease, angina/angina pectoris, heart attack, stroke?” An individual who gave a positive answer was considered a patient with CVD (17).

We used the NHANES publicly linked mortality file as of 31 December 2019, which was correlated with the NCHS with the National Death Index (NDI) through a probability matching algorithm to determine the mortality status of the patients: https://ftp.cdc.gov/pub/health_statistics/NCHS/datalinkage/linked_mortality/. The follow-up ended when an individual died or on 31 December 2019.

### Variables selection

We extracted data from the database of the following variables: age, sex, race, education level, marital status, poverty income ratio (PIR), alcohol consumption, smoking, diabetes mellitus (DM), dyslipidemia, hypertension, CKD, BMI, estimated glomerular filtration rate (eGFR), urinary albumin to creatinine ratio (UACR), HEI-2015, total energy intake, cardiovascular drugs use, cancer, arthritis, human immunodeficiency virus (HIV) infection, and glutamic oxaloacetic transaminase to serum glutamic pyruvic transaminase ratio (AST/ALT).

During the NHANES household interview, smokers were individuals who self-reported having smoked more than 100 cigarettes in their lives. The pattern of alcohol consumption was also captured by the NHANES questionnaires (18). Persons with total cholesterol (TC) ≥200 mg/dl (5.2 mmol/L), triglycerides (TG) ≥150 mg/dl (1.7 mmol/L), low-density lipoprotein cholesterol (LDL-C) ≥130 mg/dl (3.4 mmol/L), high-density lipoprotein cholesterol (HDL-C) ≤ 40 mg/dl (1.0 mmol/L), self-reported hypercholesteremia, or receiving lipid-lowering therapy were identified as patients with dyslipidemia (19). DM was defined according to a self-reported diagnosis, the use of oral hypoglycemic agents or insulin, glycosylated hemoglobin (HbAlc) ≥6.5%, a plasma glucose level ≥200 mg/dl at 2 h after the oral glucose tolerance test (OGTT), or a fasting glucose level ≥126 mg/dl. CKD was defined as eGFR <60 ml/min per 1.73 m^2^ or UACR ≥30 mg/g (20). In addition, cancer, arthritis, and HIV infections were self-reported. Dietary total energy intake was calculated through information on “total nutrient intakes” and “total dietary supplements” collected from two 24-h dietary recalls in the NHANES. BMIs were divided into four levels according to the WHO criteria, including underweight (BMI <18.5 kg/m^2^), normal weight (BMI = 18.5–24.9 kg/m^2^), overweight (BMI = 25–29.9 kg/m^2^), and obese (BMI ≥30 kg/m^2^). Participants who aged <65 years were considered middle-aged adults, whereas those who aged ≥65 years were considered older adults (21).

### Statistical analysis

Continuous data were described using mean ± standard error (mean ± SE), and a weighted *t*-test was used for comparison between the two groups. Categorical data were expressed as frequency with constituent ratio [*N* (%)] and chi-square test (χ^2^) for the comparison.

We used the MEC 2-year sample weights (WTMEC2YR) for combined analyses of NHANES data from 2007 to 2018 (22). A weighted univariate logistic regression analysis was used to screen the covariates associated with CVD, and a weighted Cox regression analysis was used to screen those associated with all-cause mortality. The associations of physical activity and sedentary behaviors with CVD in middle-aged and older patients who were overweight/obese were explored using weighted univariate and multivariate logistic regression analyses. The relationships between physical activity and sedentary behaviors and the risk of all-cause mortality were investigated using weighted univariate and multivariate Cox regression analyses (23). The interaction effects between physical activity and sedentary behaviors on CVD and the risk of all-cause mortality were also assessed. We further explored these relationships in subgroups of age and BMI.

When the study outcome was CVD, the multivariate model adjusted for age, sex, race, education level, marital status, PIR, alcohol consumption, BMI, smoking, DM, dyslipidemia, hypertension, CKD, HEI-2015, total energy intake, cardiovascular drugs use, cancer, arthritis, HIV infection, and AST/ALT. When the outcome was all-cause mortality, the multivariate model adjusted for age, sex, race, education level, marital status, PIR, BMI, smoking, DM, hypertension, CKD, total energy intake, cardiovascular drugs use, CVD, cancer, arthritis, HIV infection, and AST/ALT.

The evaluation indexes were odds ratios (ORs), hazard ratios (HRs), and 95% confidence intervals (CIs). A two-sided *P* < 0.05 was considered a significant statistical association. Statistical analyses were performed using SAS 9.4 (SAS Institute, Cary, NC, USA). The variables, including missing values, are shown in Supplementary Table S1, and they have been deleted.

## Results

### Characteristics of participants

**Figure 1:**
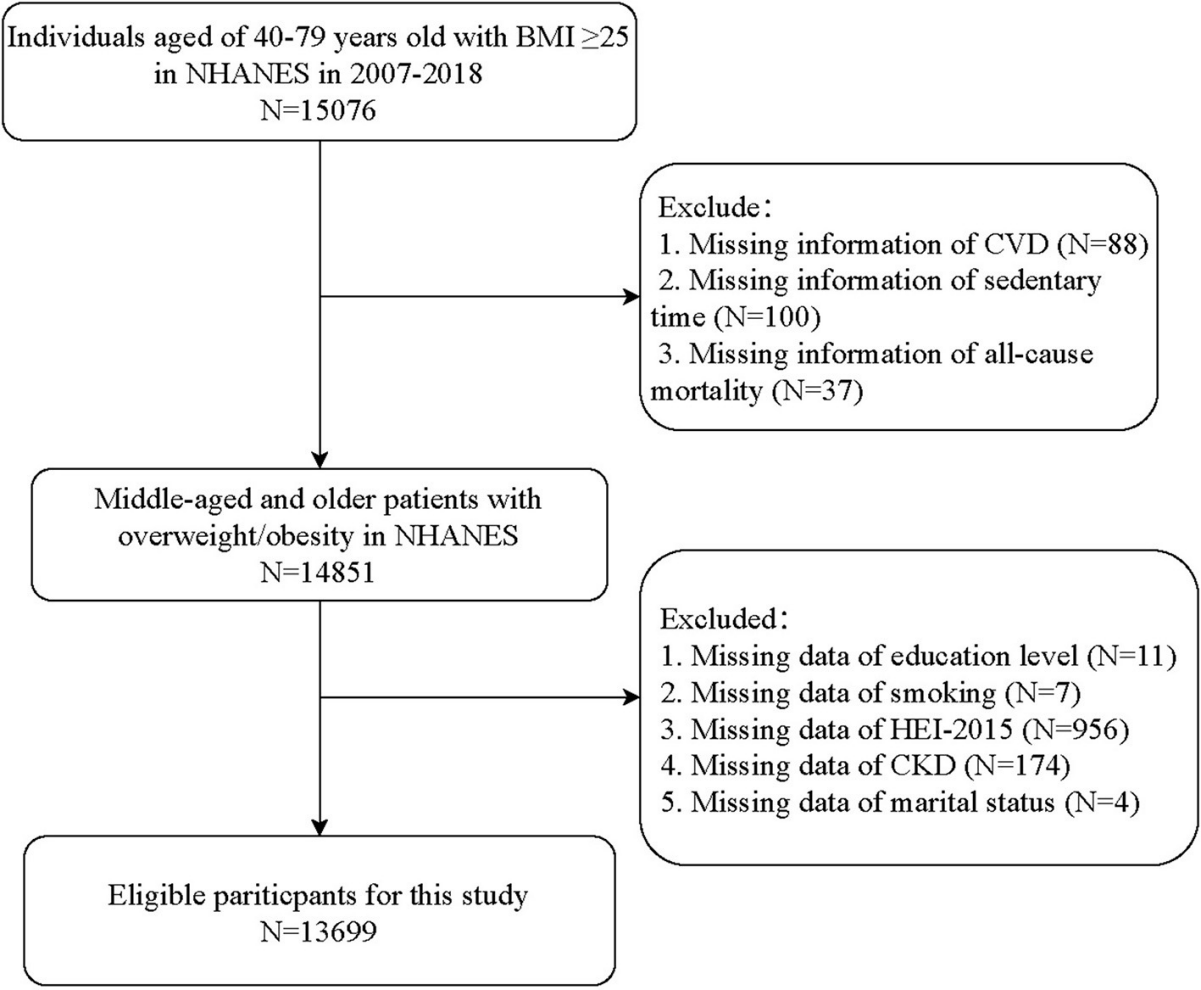
Flowchart of the participants screening.

Figure 1 shows the flowchart of participant screening. We initially included 15,076 individuals aged 40–79 years with a BMI of ≥25 kg/m^2^ from the NHANES. Then, we excluded patients without information about CVD (*n* = 88), sedentary time (*n* = 100), or all-cause mortality (*n* = 37). Those who had missing data on education level (*n* = 11), smoking (*n* = 7), HEI-2015 (*n* = 956), CKD (*n* = 174), or marital status (*n* = 4) were also excluded. Finally, 13,699 individuals were eligible.

A comparison of characteristics between the non-CVD group (*n* = 11,752) and the CVD group (*n* = 1,947) is shown in Table 1. After an average of 88.5-month follow-up, 1,560 (9.17%) participants died from all-cause. We found that 8,008 (62.96%) middle-aged and older adults were sufficiently active, whereas 6,577 (54.19%) had high sedentary time.

**Table 1:** Characteristics of middle-aged and older adults with overweight/obesity.

**Variables**	**Total (*n* = 13,699)**	**Non-CVD (*n* = 11,752)**	**CVD (*n* = 1,947)**	**Statistics**	***P*-value**
**Age, years**, ***n*** **(%)**				χ^2^ = 379.667	<0.001
<65	9,738 (75.92)	8,799 (79.33)	939 (50.79)		
≥65	3,961 (24.08)	2,953 (20.67)	1,008 (49.21)		
**Sex**, ***n*** **(%)**				χ^2^ = 29.872	<0.001
Male	6,763 (50.83)	5,627 (49.69)	1,136 (59.23)		
Female	6,936 (49.17)	6,125 (50.31)	811 (40.77)		
**Race**, ***n*** **(%)**				χ^2^ = 15.546	0.004
Mexican American	2,339 (7.67)	2,103 (7.98)	236 (5.43)		
Other Hispanic	1,618 (5.26)	1,421 (5.39)	197 (4.31)		
Non-Hispanic White	5,495 (70.53)	4,575 (70.35)	920 (71.83)		
Non-Hispanic Black	3,224 (11.21)	2,743 (11.07)	481 (12.23)		
Other Race—including multi-racial	1,023 (5.33)	910 (5.21)	113 (6.20)		
**Education level**, ***n*** **(%)**				χ^2^ = 64.545	<0.001
Less than 9th grade	1,781 (5.96)	1,487 (5.65)	294 (8.23)		
9–11th grade	1,910 (9.96)	1,577 (9.57)	333 (12.80)		
High school graduate/GED or equivalent	3,153 (24.08)	2,637 (23.44)	516 (28.78)		
Some college or AA	3,966 (31.42)	3,414 (31.55)	552 (30.46)		
College graduate or above	2,889 (28.58)	2,637 (29.78)	252 (19.73)		
**Marital status**, ***n*** **(%)**				χ^2^ = 52.128	<0.001
Married	8,032 (64.71)	6,986 (65.50)	1,046 (58.88)		
Widowed	1,143 (6.17)	894 (5.54)	249 (10.85)		
Divorced	2,046 (14.08)	1,714 (13.93)	332 (15.17)		
Separated	562 (2.54)	483 (2.48)	79 (2.97)		
Never married	1,209 (7.65)	1,056 (7.77)	153 (6.80)		
Living with partner	707 (4.85)	619 (4.79)	88 (5.33)		
**PIR**, ***n*** **(%)**				χ^2^ = 49.269	<0.001
≤ 1	2,300 (9.92)	1,835 (9.17)	465 (15.47)		
>1	10,062 (82.51)	8,754 (83.29)	1,308 (76.79)		
Unknown	1,337 (7.57)	1,163 (7.54)	174 (7.74)		
**Alcohol consumption**, ***n*** **(%)**				χ^2^ = 10.049	0.007
Yes	3,689 (26.96)	3,111 (26.36)	578 (31.39)		
No	1,795 (9.48)	1,585 (9.63)	210 (8.32)		
Unknown	8,215 (63.56)	7,056 (64.00)	1,159 (60.29)		
**Smoking**, ***n*** **(%)**				χ^2^ = 104.034	<0.001
Yes	7,237 (52.67)	6,510 (54.85)	727 (36.64)		
No	6,462 (47.33)	5,242 (45.15)	1,220 (63.36)		
**DM**, ***n*** **(%)**				χ^2^ = 356.999	<0.001
Yes	3,647 (20.81)	2,762 (18.10)	885 (40.72)		
No	10,052 (79.19)	8,990 (81.90)	1,062 (59.28)		
**Dyslipidemia**, ***n*** **(%)**				χ^2^ = 89.357	<0.001
Yes	11,060 (81.79)	9,319 (80.48)	1,741 (91.49)		
No	2,263 (16.00)	2,090 (17.19)	173 (7.25)		
Unknown	376 (2.21)	343 (2.34)	33 (1.26)		
**Hypertension**, ***n*** **(%)**				χ^2^ = 253.291	<0.001
Yes	10,020 (69.47)	8,190 (66.25)	1,830 (93.18)		
No	36,79 (30.53)	3,562 (33.75)	117 (6.82)		
**CKD**, ***n*** **(%)**				χ^2^ = 253.978	<0.001
Yes	817 (4.56)	500 (3.36)	317 (13.38)		
No	12,882 (95.44)	11,252 (96.64)	1,630 (86.62)		
**BMI**, ***n*** **(%)**				χ^2^ = 59.311	<0.001
<30	6,165 (45.84)	5,437 (47.20)	728 (35.85)		
≥30	7,534 (54.16)	6,315 (52.80)	1,219 (64.15)		
eGFR, mean (SE)	92.39 (0.28)	93.77 (0.31)	82.26 (0.55)	*t* = 18.54	<0.001
UACR, mean (SE)	0.38 (0.03)	0.33 (0.03)	0.80 (0.11)	*t* = −4.41	<0.001
HEI-2015, mean (SE)	54.08 (0.22)	54.23 (0.23)	52.95 (0.46)	*t* = 2.67	0.009
Total energy intake, kcal, mean (SE)	2,065.99 (10.59)	2,084.50 (11.49)	1,929.77 (25.57)	*t* = 5.45	<0.001
**Cardiovascular drugs use**, ***n*** **(%)**				χ^2^ = 378.996	<0.001
Yes	2,951 (19.50)	2,094 (16.65)	857 (40.46)		
No	10,748 (80.50)	9,658 (83.35)	1,090 (59.54)		
**Cancer**, ***n*** **(%)**				χ^2^ = 48.822	<0.001
Yes	1,604 (13.35)	1,245 (12.39)	359 (20.43)		
No	12,084 (86.56)	10,500 (87.55)	1,584 (79.26)		
Unknown	11 (0.08)	7 (0.05)	4 (0.31)		
**Arthritis**, ***n*** **(%)**				χ^2^ = 149.848	<0.001
Yes	5,314 (38.10)	4,182 (35.53)	1,132 (57.04)		
No	8,358 (61.75)	7,545 (64.32)	813 (42.83)		
Unknown	27 (0.15)	25 (0.15)	2 (0.13)		
**HIV infection**, ***n*** **(%)**				χ^2^ = 189.946	<0.001
Yes	23 (0.13)	20 (0.13)	3 (0.11)		
No	4,216 (34.75)	3,921 (37.14)	295 (17.13)		
Unknown	9,460 (65.12)	7,811 (62.73)	1,649 (82.76)		
AST/ALT, mean (SE)	1.06 (0.00)	1.05 (0.00)	1.10 (0.01)	*t* = −5.02	<0.001
**Physical activity**, ***n*** **(%)**				χ^2^ = 53.069	<0.001
Sufficiently active	8,008 (62.96)	7,068 (64.39)	940 (52.44)		
Insufficiently active	5,691 (37.04)	4,684 (35.61)	1,007 (47.56)		
**Sedentary behaviors**, ***n*** **(%)**				χ^2^ = 12.265	<0.001
Low sedentary time	7,122 (45.81)	6,255 (46.47)	867 (40.94)		
High sedentary time	6,577 (54.19)	5,497 (53.53)	1,080 (59.06)		
No	12,139 (90.83)	10,622 (92.06)	1,517 (81.79)		
Yes	1,560 (9.17)	1,130 (7.94)	430 (18.21)		
Follow-up time, months, mean (SE)	88.50 (1.54)	89.45 (1.61)	81.48 (2.16)	*t* = 3.75	<0.001

t, t-test; χ^2^, chi-square test.

CVD, cardiovascular disease; PIR, poverty income ratio; DM, diabetes mellitus; CKD, chronic kidney disease; BMI, body mass index; eGFR, estimated glomerular filtration rate; SE, standard error; UACR, urinary albumin to creatinine ratio; HEI-2015, healthy eating index 2015; AST, glutamic oxaloacetic transaminase; ALT, glutamic pyruvic transaminase GED, general educational development; AA, associate degree.

### Associations of physical activity and sedentary behaviors with CVD and all-cause mortality, respectively

We first screened the covariates associated with CVD and all-cause mortality, respectively (Supplementary Table S2). Then, we respectively explored the relationships of physical activity and sedentary behaviors with CVD (Table 2) and of physical activity with sedentary behaviors and all-cause mortality (Table 3). After adjusting for covariates, we found that compared with low sedentary time, patients who had a higher sedentary time seemed to have both higher odds of CVD [OR = 1.24, 95% CI: (1.06–1.44)] and a higher risk of all-cause mortality [HR = 1.20, 95% CI: (1.06–1.37)]. Furthermore, being insufficiently active was linked to both high odds of CVD [OR = 1.24, 95% CI: (1.05–1.46)] and a high risk of all-cause mortality [HR = 1.32, 95% CI: (1.15–1.51)] among overweight or obese middle-aged and older adults.

**Table 2:** Association between physical activity, sedentary behaviors, and CVDs.

**Variable**	**Crude model**		**Adjusted model** ^*^	
	**OR (95% CI)**	***P*** **-value**	**OR (95% CI)**	***P*** **-value**
Low sedentary time	Ref		Ref	
High sedentary time	1.25 (1.10–1.42)	<0.001	1.21 (1.06–1.39)	0.006
Sufficiently active	Ref		Ref	
Insufficiently active	1.64 (1.43–1.88)	<0.001	1.58 (1.41–1.76)	<0.001

CVD, cardiovascular disease; OR, odds ratio; CI, confidence interval; Ref, reference.

^*^Adjusted for age, sex, race, education level, marital status, PIR, alcohol consumption, BMI, smoking, DM, dyslipidemia, hypertension, CKD, HEI-2015, total energy intake, cardiovascular drugs use, cancer, arthritis, HIV infection, and AST/ALT.

**Table 3:** Association between physical activity, sedentary behaviors, and all-cause mortality.

**Variable**	**Crude model**		**Adjusted model** ^*^	
	**HR (95% CI)**	***P*** **-value**	**HR (95% CI)**	***P*** **-value**
Low sedentary time	Ref		Ref	
High sedentary time	1.24 (1.06–1.44)	0.006	1.20 (1.06–1.37)	0.006
Sufficiently active	Ref		Ref	
Insufficiently active	1.24 (1.05–1.46)	0.011	1.32 (1.15–1.51)	<0.001

CVD, cardiovascular disease; HR, hazard ratio; CI, confidence interval; Ref, reference.

^*^Adjusted for age, sex, race, education level, marital status, PIR, BMI, smoking, DM, hypertension, CKD, total energy intake, cardiovascular drugs use, CVD, cancer, arthritis, HIV infection, and AST/ALT.

### Interaction effect between physical activity and sedentary behaviors on CVD and all-cause mortality

We further investigated the interaction effects between physical activity and sedentary behaviors on CVD and all-cause mortality (Table 4). After adjusting for covariates, the results showed that middle-aged and older adults who were overweight/obese had high sedentary time combined with insufficient physical activity and seemed to have both high odds of CVD [OR = 1.44, 95% CI: (1.20–1.73)] and a high risk of all-cause mortality [HR = 1.48, 95% CI: (1.24–1.76)], compared with those who had low sedentary time combined with sufficient physical activity.

**Table 4:** Interaction between physical activity, sedentary behaviors in CVD, and all-cause mortality.

**Interaction**	**CVD** ^**#**^		**All-cause mortality** ^*^	
	**OR (95% CI)**	***P*** **-value**	**HR (95% CI)**	***P*** **-value**
Low sedentary time and sufficiently active	Ref		Ref	
Low sedentary time and insufficiently active	1.01 (0.78–1.31)	0.948	1.10 (0.90–1.33)	0.358
High sedentary time and sufficiently active	1.07 (0.87–1.31)	0.543	1.02 (0.84–1.24)	0.838
High sedentary time and insufficiently active	1.44 (1.20–1.73)	<0.001	1.48 (1.24–1.76)	<0.001

CVD, cardiovascular disease; OR, odds ratio; HR, hazard ratio; CI, confidence interval; Ref, reference.

^#^Adjusted for age, sex, race, education level, marital status, PIR, alcohol consumption, BMI, smoking, DM, dyslipidemia, hypertension, CKD, HEI-2015, total energy intake, cardiovascular drugs use, cancer, arthritis, HIV infection, and AST/ALT.

^*^Adjusted for age, sex, race, education level, marital status, PIR, BMI, smoking, DM, hypertension, CKD, total energy intake, cardiovascular drugs use, CVD, cancer, arthritis, HIV infection, and AST/ALT.

### The combined effects of physical activity with sedentary behaviors on CVD and all-cause mortality in age and BMI subgroups

Table 5 shows the subgroup analyses of the combined effect between physical activity and sedentary behaviors on CVD and all-cause mortality. For patients aged ≥65 years, high sedentary time [OR = 1.41, 95% CI: (1.11–1.78)] and being insufficiently active [OR = 1.25, 95% CI: (1.02–1.54)] were both associated with high odds of CVD, whereas these relationships in the <65-year-old subgroup were not significant (all *P* > 0.05). Similarly, in the BMI ≥30 kg/m^2^ group, patients with high sedentary time [OR = 1.29, 95% CI: (1.02–1.61)] or who were insufficiently active [OR = 1.27, 95% CI: (1.04–1.57)] had high odds of CVD, while these associations were not significant among those whose BMI was <30 kg/m^2^ (all *P* > 0.05). In addition, the interaction effect between physical activity and sedentary behaviors on CVD was found in patients in the subgroups of <65 years, ≥65 years, and with a BMI of ≥30 kg/m^2^ (all *P* < 0.05).

**Table 5:** Association between physical activity, sedentary behaviors, CVD, and all-cause mortality in age and BMI subgroups.

**Variable**	**CVD** ^**#**^		**All-cause mortality** ^*^	
	**OR (95% CI)**	***P*** **-value**	**HR (95% CI)**	***P*** **-value**
**Age**<**65**				
Low sedentary time	Ref		Ref	
High sedentary time	1.13 (0.93–1.36)	0.222	1.13 (0.89–1.42)	0.313
Sufficiently active	Ref		Ref	
Insufficiently active	1.19 (0.95–1.49)	0.13	1.15 (0.98–1.36)	0.094
Low sedentary time and sufficiently active	Ref		Ref	
Low sedentary time and insufficiently active	1.03 (0.73–1.44)	0.877	1.02 (0.75–1.39)	0.895
High sedentary time and sufficiently active	1.01 (0.77–1.32)	0.967	1.04 (0.78–1.38)	0.805
High sedentary time and insufficiently active	1.29 (1.01–1.65)	0.043	1.26 (0.96–1.63)	0.091
**Age** ≥**65**				
Low sedentary time	Ref		Ref	
High sedentary time	1.41 (1.11–1.78)	0.005	1.34 (1.12–1.60)	0.001
Sufficiently active	Ref		Ref	
Insufficiently active	1.25 (1.02–1.54)	0.034	1.51 (1.22–1.87)	<0.001
Low sedentary time and sufficiently active	Ref		Ref	
Low sedentary time and insufficiently active	0.98 (0.72–1.35)	0.916	1.12 (0.85–1.48)	0.421
High sedentary time and sufficiently active	1.19 (0.87–1.63)	0.265	0.97 (0.75–1.27)	0.843
High sedentary time and insufficiently active	1.61 (1.20–2.17)	0.002	1.76 (1.37–2.27)	<0.001
**BMI**<**30**				
Low sedentary time	Ref		Ref	
High sedentary time	1.10 (0.88–1.39)	0.394	1.23 (0.97–1.56)	0.081
Sufficiently active	Ref		Ref	
Insufficiently active	1.07 (0.84–1.38)	0.572	1.46 (1.17–1.83)	<0.001
Low sedentary time and sufficiently active	Ref		Ref	
Low sedentary time and insufficiently active	0.82 (0.55–1.23)	0.342	1.14 (0.85–1.54)	0.383
High sedentary time and sufficiently active	0.93 (0.68–1.26)	0.628	0.99 (0.72–1.35)	0.934
High sedentary time and insufficiently active	1.22 (0.89–1.67)	0.219	1.72 (1.26–2.35)	<0.001
**BMI** ≥**30**				
Low sedentary time	Ref		Ref	
High sedentary time	1.29 (1.02–1.61)	0.032	1.19 (1.03–1.37)	0.017
Sufficiently active	Ref		Ref	
Insufficiently active	1.27 (1.04–1.57)	0.022	1.18 (0.97–1.43)	0.097
Low sedentary time and sufficiently active	Ref		Ref	
Low sedentary time and insufficiently active	1.10 (0.80–1.51)	0.554	1.02 (0.76–1.37)	0.907
High sedentary time and sufficiently active	1.15 (0.85–1.56)	0.369	1.06 (0.81–1.39)	0.661
High sedentary time and insufficiently active	1.51 (1.17–1.94)	0.002	1.30 (1.06–1.61)	0.013

CVD, cardiovascular disease; BMI, body mass index; OR, odds ratio; HR, hazard ratio; CI, confidence interval; Ref, reference.

^#^Adjusted for age (not adjusted in age subgroup), sex, race, education level, marital status, PIR, alcohol consumption, BMI (not adjusted in BMI subgroup), smoking, DM, dyslipidemia, hypertension, CKD, HEI-2015, total energy intake, cardiovascular drugs use, cancer, arthritis, HIV infection, and AST/ALT.

^*^Adjusted for age (not adjusted in age subgroup), sex, race, education level, marital status, PIR, BMI (not adjusted in BMI subgroup), smoking, DM, hypertension, CKD, total energy intake, cardiovascular drugs use, CVD, cancer, arthritis, HIV infection, and AST/ALT.

High sedentary time was linked to a high risk of all-cause mortality in patients aged ≥65 years [HR = 1.34, 95% CI: (1.12–1.60)]. Being insufficiently active was associated with a high risk of all-cause mortality in patients aged ≥65 years [HR = 1.51, 95% CI: (1.22–1.87)] and with BMI <30 kg/m^2^ [HR = 1.46, 95% CI: (1.17–1.83)]. Moreover, the interaction effect between high sedentary time and being insufficiently active on having a high risk of all-cause mortality was found in patients aged ≥65 years, with BMI <30 kg/m^2^, and BMI ≥30 kg/m^2^ subgroups (all *P* < 0.05).

## Discussion

In this study, we explored the relationships between physical activity and sedentary behaviors with CVD and all-cause mortality risk in middle-aged and older patients who were overweight/obese, respectively. We also assessed the interaction effects between physical activity and sedentary behaviors on CVD as well as all-cause mortality. The results showed that high sedentary time and being insufficiently active had an interaction effect on both high odds of CVD and a high risk of all-cause mortality. These associations were also found in patients with different ages and BMI levels.

To the best of our knowledge, no study has explored the interaction effect between physical activity and sedentary behaviors on CVD and all-cause mortality in middle-aged and older adults who are overweight or obese. A prospective cohort study in 70-year-old populations showed that objectively measured light-intensity physical activity and moderate-intensity physical activity were each associated with a lower risk of all-cause mortality, while sedentary time was associated with increased risk (24). Liang et al. (25) found that long-term sedentary behaviors increased the risk of CVDs in healthy adults, whereas physical activity reduced the risk of CVDs and improved indicators associated with CVDs. Our findings relatively further verified these relationships in middle-aged and older adults who were overweight/obese, and differently, we based them on the NHANES database, which includes representative populations in the United States. Some studies indicated a potential regulating effect of physical activity on the sedentary behaviors associated with CVD or all-cause mortality risk. However, they often focus on older populations or obese populations. Han et al. (26) considered that despite increased time spent on sedentary behaviors, maintaining physical activity could reduce the occurrence of CVD in healthy Korean adults. Furthermore, physical activity and sedentary behaviors are associated with adiposity-related traits, apparently in a bidirectional manner (27). Therefore, the current study combined their study populations and investigated and suggested that high sedentary time combined with low physical activity was linked to a high risk of both CVD and all-cause mortality in middle-aged and older patients who were overweight/obese.

The mechanisms through which sedentary behaviors combined with physical activity influence CVD and all-cause mortality are complex and multifaceted. Sedentary behaviors have been identified as a risk factor for CVD, which was suggested to have the strongest association with all causes of CVD mortality (28, 29). Sedentary behaviors result in a lack of physical activity, leading to a decreased turnover of endogenous energy stores, myogenic glycogen, and intracellular lipids, which in turn can lead to skeletal muscle insulin resistance (25). The increased adipogenesis promotes the production of very low-density lipoproteins and lower high-density lipoprotein levels in the liver in hyperinsulinism, which may contribute to the development of metabolic syndrome and also CVDs. Additionally, steatosis may cause hyperglycemia, which not only causes DM but also potentially increases the risk of CVD (30, 31). Several behavioral and biological pathways could explain the potential regulating effect of physical activity on CVD and the all-cause mortality risk associated with sedentary behaviors, including metabolic and sex hormones, inflammation, and immunity (32). Prolonged, uninterrupted sitting is related to impaired glucose metabolism and increased systemic inflammation, which can be attenuated by physical activities (33, 34). Moreover, sitting may induce exercise resistance and diminish the benefits of physical activity (35). Nevertheless, additional studies are needed to evaluate whether the observed interaction between sedentary behaviors and physical activity on CVD and all-cause mortality is causal.

The joint association between sedentary behaviors and physical activity on CVD and mortality outcomes has been explored in healthy populations. A harmonized meta-analysis summarized evidence from 13 studies of more than 1 million adults and showed that long sedentary time was associated with increased all-cause mortality among adults who engaged in <30 MET hour/week of physical activity (11). In 2018, the United States Department of Health and Human Services clearly acknowledged the health risks associated with sedentary behavior and suggested that people of all ages would benefit from more regular physical activity and less sedentary behavior for the first time in the Physical Activity Guidelines for Americans (36). Despite the limited evidence available, the WHO strongly recommends limiting the amount of sedentary time and substituting it with any physical activity to improve health, particularly for individuals with long-term diseases (10).

We also explored the associations of sedentary behaviors and physical activity with CVD and all-cause mortality risk in individuals of different ages and BMIs. The subgroup analysis results showed that the combined effect of physical activity and sedentary behaviors on CVD was significant in patients aged <65 and ≥65 years, with BMI ≥30 kg/m^2^. Furthermore, this interaction on risk of all-cause mortality was found in patients in the subgroups aged ≥65 years with BMI <30 kg/m^2^ and BMI ≥30 kg/m^2^. Physical activity is a protective factor of successful aging in middle-aged and older populations, which can prevent the development of many chronic diseases, such as metabolic syndrome, DM, hypertension, dyslipidemia, depression, osteoarthritis, osteoporosis, and non-alcoholic fatty liver disease (37). In fact, there is a dose-response relationship between physical activity and a decrease in mortality in middle-aged and older people (38). In the current research, we found that long sedentary time combined with low physical activity levels was associated with high odds of CVD in overweight/obese adults aged <65 years, while this combined effect was associated with a high risk of all-cause mortality in those aged ≥65 years. It was inferred that middle-aged people who were overweight or obese may pay more attention to improving and preventing the development of CVD because their basic health condition may be better than that of older people. Likewise, older adults should take care of their sedentary time and increase appropriate physical activity to reduce the risk of mortality. Moreover, in patients with obesity (BMI ≥30 kg/m^2^), high sedentary time as well as low physical activity may increase the risk of both CVD and all-cause mortality in our study. Clark et al. (39) concluded that total physical activity habits, including time spent being sedentary and lower-intensity physical activity, impact the levels of lipoprotein-associated phospholipase A2, an important inflammatory marker and marker of CVD risk. Wanders et al. (40) confirmed a beneficial association between physical activity and whole-body insulin resistance. These findings indicated that the potential mechanisms of the relationship between sedentary behaviors and physical activity, CVD, and all-cause mortality may be varied. Therefore, the causal associations and specific mechanisms between sedentary behaviors, physical activity, and CVD/mortality need to be clarified in the future, which may be beneficial management and preventive measures for chronic disease and mortality in obese patients.

This study is based on the NHANES database, which has large samples with good representativeness in the United States. We analyzed the combined effect between physical activity and sedentary behaviors on CVD and all-cause mortality risk, which may provide some references for behavioral interventions in overweight/obese middle-aged and older adults (e.g., appropriate physical activity to antagonize the risks associated with prolonged sedentary time). However, there are also some limitations to our study. The information about CVD was collected according to self-reported diagnosis, and that of physical activity as well as sedentary behaviors was obtained through questionnaires, which cannot avoid the reporting bias. Additionally, although we have tried our best to include possible confounding factors, such as diet, lifestyle, comorbidities, and treatment, due to the limitations of the NHANES database, other potential covariates, including place of abode (urban or rural), changes in lifestyle, and treatment regimen during the follow-up, could not be obtained and included in this exploration.

## Conclusion

Low physical activity and long sedentary time were associated with both high odds of CVDs and a high risk of all-cause mortality in middle-aged and older adults who were overweight or obese, and there was an interaction effect between them.

## Data availability statement

Publicly available datasets were analyzed in this study. This data can be found at: NHANES database, https://wwwn.cdc.gov/nchs/nhanes/.

## Ethics statement

The requirement of ethical approval was waived by the People's Hospital of Lincang for the studies involving humans because the database is publicly available. The studies were conducted in accordance with the local legislation and institutional requirements. The participants provided their written informed consent to participate in this study.

## Author contributions

YZ: Conceptualization, Data curation, Formal analysis, Investigation, Methodology, Project administration, Supervision, Writing—original draft, Writing—review & editing. XL: Writing—original draft, Writing—review & editing.
