# Supplementary material for: Effects of physical activity and sedentary behaviors on cardiovascular disease and the risk of all-cause mortality in overweight or obese middle-aged and older adults
Source: Front Public Health. 2024 Feb 12;12:1302783. doi: 10.3389/fpubh.2024.1302783 (PMC10894908; doi:10.3389/fpubh.2024.1302783)
Supplement: Supplementary file 1 [file Table_1.docx]

**Table S1. Missing data and proportions**

| Variables | n (%) |
| --- | --- |
| Education level | 11 (0.07) |
| Marital status | 7 (0.05) |
| Total energy intake | 959 (6.46) |
| cardiovascular drugs use | 0 (0.00) |
| HEI-2015 | 959 (6.46) |
| CKD | 211 (1.42) |

HEI-2015: healthy eating index-2015, CKD: chronic kidney disease

**Table S2. Covariates for CVD and for all-cause mortality**

| Variables | CVD | | All-cause mortality | |
| --- | --- | --- | --- | --- |
|  | OR (95% CI) | *P* | HR (95% CI) | *P* |
| Age |  |  |  |  |
| <65 | Ref |  | Ref |  |
| ≥65 | 3.72 (3.24-4.26) | <0.001 | 2.61 (2.26-3.02) | <0.001 |
| Gender |  |  |  |  |
| Male | Ref |  | Ref |  |
| Female | 0.68 (0.59-0.78) | <0.001 | 0.82 (0.72-0.94) | 0.003 |
| Race |  |  |  |  |
| Mexican American | Ref |  | Ref |  |
| Other Hispanic | 1.18 (0.93-1.48) | 0.170 | 0.96 (0.74-1.25) | 0.775 |
| Non-Hispanic White | 1.50 (1.23-1.83) | <0.001 | 1.32 (1.12-1.57) | 0.001 |
| Non-Hispanic Black | 1.62 (1.32-1.99) | <0.001 | 1.35 (1.17-1.55) | <0.001 |
| Other race-including multi-racial | 1.75 (1.18-2.59) | 0.006 | 1.22 (0.85-1.76) | 0.281 |
| Education level |  |  |  |  |
| Less than 9th grade | Ref |  | Ref |  |
| 9-11th grade | 0.92 (0.73-1.16) | 0.463 | 0.78 (0.60-1.01) | 0.063 |
| High school Graduate/GED or equivalent | 0.84 (0.67-1.06) | 0.149 | 0.68 (0.53-0.88) | 0.004 |
| Some college or AA degree | 0.66 (0.55-0.80) | <0.001 | 0.66 (0.51-0.85) | 0.001 |
| College graduate or above | 0.45 (0.35-0.59) | <0.001 | 0.50 (0.40-0.62) | <0.001 |
| Marital status |  |  |  |  |
| Married | Ref |  | Ref |  |
| Widowed | 2.18 (1.76-2.70) | <0.001 | 2.26 (1.89-2.71) | <0.001 |
| Divorced | 1.21 (0.98-1.50) | 0.076 | 1.35 (1.13-1.61) | <0.001 |
| Separated | 1.33 (0.96-1.86) | 0.087 | 1.42 (1.03-1.96) | 0.033 |
| Never married | 0.97 (0.74-1.28) | 0.846 | 1.22 (0.90-1.66) | 0.206 |
| Living with partner | 1.24 (0.90-1.70) | 0.179 | 1.18 (0.86-1.61) | 0.298 |
| PIR |  |  |  |  |
| ≤1 | Ref |  | Ref |  |
| >1 | 0.55 (0.47-0.63) | <0.001 | 0.59 (0.50-0.70) | <0.001 |
| Unknown | 0.61 (0.45-0.82) | 0.001 | 0.58 (0.43-0.76) | <0.001 |
| Drinking |  |  |  |  |
| No | Ref |  | Ref |  |
| Yes | 1.38 (1.10-1.74) | 0.007 | 1.14 (0.92-1.41) | 0.230 |
| Unknown | 1.09 (0.87-1.36) | 0.442 | 1.00 (0.82-1.23) | 0.964 |
| BMI |  |  |  |  |
| <30 | Ref |  | Ref |  |
| ≥30 | 1.60 (1.41-1.81) | <0.001 | 1.24 (1.10-1.39) | <0.001 |
| Smoking |  |  |  |  |
| No | Ref |  | Ref |  |
| Yes | 2.10 (1.82-2.43) | <0.001 | 1.75 (1.53-2.00) | <0.001 |
| DM | |  |  |  |
| No | Ref |  | Ref |  |
| Yes | 3.11 (2.74-3.53) | <0.001 | 2.07 (1.84-2.32) | <0.001 |
| Dyslipidemia | |  |  |  |
| No | Ref |  | Ref |  |
| Yes | 2.69 (2.16-3.36) | <0.001 | 1.14 (0.93-1.38) | 0.203 |
| Unknown | 1.28 (0.74-2.19) | 0.373 | 1.16 (0.82-1.65) | 0.396 |
| Hypertension |  |  |  |  |
| No | Ref |  | Ref |  |
| Yes | 6.96 (5.27-9.19) | <0.001 | 1.76 (1.45-2.13) | <0.001 |
| CKD |  |  |  |  |
| No | Ref |  | Ref |  |
| Yes | 4.44 (3.61-5.47) | <0.001 | 3.33 (2.73-4.06) | <0.001 |
| HEI-2015 | 0.90 (0.84-0.98) | 0.01 | 0.94 (0.87-1.00) | 0.066 |
| Total energy intake | 0.81 (0.75-0.88) | <0.001 | 0.89 (0.82-0.96) | 0.002 |
| Cardiovascular drugs use |  |  |  |  |
| No | Ref |  | Ref |  |
| Yes | 3.40 (2.96-3.91) | <0.001 | 1.87 (1.64-2.12) | <0.001 |
| CVD |  |  |  |  |
| No |  |  | Ref |  |
| Yes |  |  | 2.58 (2.26-2.95) | <0.001 |
| Cancer |  |  |  |  |
| Yes | Ref |  | Ref |  |
| No | 0.55 (0.44-0.68) | <0.001 | 0.59 (0.50-0.70) | <0.001 |
| Unknown | 3.46 (0.92-13.03) | 0.066 | 0.00 (0.00-0.00) | <0.001 |
| Arthritis |  |  |  |  |
| Yes | Ref |  | Ref |  |
| No | 0.41 (0.35-0.49) | <0.001 | 0.69 (0.60-0.81) | <0.001 |
| Unknown | 0.55 (0.09-3.28) | 0.511 | 1.19 (0.22-6.26) | 0.841 |
| HIV infection |  |  |  |  |
| Yes | Ref |  | Ref |  |
| No | 0.56 (0.15-2.09) | 0.384 | 0.60 (0.12-2.87) | 0.518 |
| Unknown | 1.60 (0.42-6.06) | 0.483 | 1.23 (0.25-5.95) | 0.797 |
| AST/ALT | 1.17 (1.10-1.24) | <0.001 | 1.23 (1.14-1.32) | <0.001 |

CVD: cardiovascular disease, OR: odds ratio, CI: confidence interval, Ref: reference, HR: hazard ratio, PIR: poverty income ratio, BMI: body mass index, DM: diabetes mellitus, CKD: chronic kidney disease, HEI-2015: healthy eating index-2015, AST: glutamic oxaloacetic transaminase, ALT: glutamic pyruvic transaminase.
